# Reduced fertilization supplemented with *Bacillus safensis* RGM 2450 and *Bacillus siamensis* RGM 2529 promotes tomato production in a sustainable way

**DOI:** 10.3389/fpls.2024.1451887

**Published:** 2024-08-22

**Authors:** Fabiola Altimira, Sebastián Godoy, Matías Arias-Aravena, Nataly Vargas, Erick González, Elena Dardón, Edgar Montenegro, Ignacio Viteri, Eduardo Tapia

**Affiliations:** ^1^ Laboratorio de Entomología y Biotecnología, Instituto de Investigaciones Agropecuarias, INIA La Platina, Santiago, Chile; ^2^ Laboratorio de Biotecnología, Centro de Excelencia Microbiano, El Jocotillo, Guatemala, Guatemala

**Keywords:** *Bacillus safensis*, *Bacillus siamensis*, tomato, fertilization, plant growth-promoting rhizobacteria

## Abstract

The rising demand for vegetables has driven the adoption of greenhouse cultivation to guarantee high yields and quality of fresh produce year-round. Consequently, this elevates the demand for fertilizers, whose costs are progressively escalating. *Bacillus safensis* RGM 2450 and *Bacillus siamensis* RGM 2529 are plant growth-promoting rhizobacteria (PGPR). The combination of these strains exhibited synergistic activity in stimulating the growth and seedling hydration of tomatoes. In this study, the effects of inoculation with a RGM 2450 plus RGM 2529 formulation were evaluated under 66% and 100% fertilization programs in tomato crops under greenhouse conditions. Fertilization programs (66% and 100%) with or without commercial biostimulants were used as control treatments. In this assay, the NPK percentage in the plant tissue, tomato average weight, tomato average weight per harvest, tomato diameter, and changes in the colonization, structure, and diversity of the bacterial rhizosphere were measured. The 100% and 66% fertilization programs supplemented with the RGM 2529 plus RGM 2450 formulation increased the average weight of tomatoes per harvest without statistical difference between them, but with the other treatments. The 66% fertilization with RGM 2450 plus RGM 2529 increased between 1.5 and 2.0 times the average weight of tomatoes per harvest compared to the 66% and 100% fertilizations with and without commercial biostimulant treatments, respectively. This study represents the first report demonstrating that the application of a formulation based on a mixture of *B. siamensis* and *B. safensis* in a fertilization program reduced by 33% is equivalent in productivity to a conventional fertilization program for tomato cultivation, achieving an increase in potential plant growth-promoting rizobacteria of the genus *Flavobacterium*. Therefore, the adoption of a combination of these bacterial strains within the framework of a 66% inorganic fertilization program is a sustainable approach to achieving greater tomato production and reducing the environmental risks associated with the use of inorganic fertilization.

## 1 Introduction

Tomatoes are among the most consumed vegetable crops worldwide (Food and Agriculture Organization of the United Nations (FAO), 2018). The harvested area of tomatoes covers approximately 5 million hectares and generates approximately 180 million tons annually (Altimira et al., 2022; Food and Agriculture Organization of the United Nations (FAO), 2023). The main tomato-producing country is China, followed by the United States, India, members of the European Union, and Turkey. These countries together supply approximately 70% of global tomato production (Food and Agriculture Organization of the United Nations (FAO), 2023).

Tomato cultivation is carried out both in open field and in greenhouses. However, worldwide, greenhouse cultivation has experienced significant growth in recent decades (Aznar-Sánchez et al., 2020). A recent estimate quantifies the global coverage at 1.3 million hectares covered by greenhouses in 119 countries on five continents, demonstrating that greenhouse cultivation represents a global phenomenon (Tong et al., 2024). Approximately 50% of the total tomato production in China is carried out in greenhouses (The Science Agriculture, 2024; Tong et al., 2024). The increasing adoption in production under protected conditions is attributed to the ability of these systems to cope with relevant challenges such as climate change and pest control, allowing for more efficient year-round production (Goddek et al., 2023; Barreca, 2024). Tomato cultivation requires essential macronutrients such as nitrogen (N), phosphorus (P), potassium (K), and calcium (Ca) for its physiological functioning and the complete development of its vegetative cycle (Marschner, 2012). These nutrients are typically supplied by inorganic fertilizers. The high-frequency and disproportionate application of this type of fertilizer increases the amount of salt in the soil (Ilahi et al., 2021). This increase in salinity reduces water absorption by plants and causes crop tip browning, lower leaf yellowing, leaf bending, and crop lodging (Ilahi et al., 2021). Additionally, inorganic fertilizers contain large amounts of hydrochloric acid, sulfuric acid, and phosphoric acid. These acids lead to soil acidification, which can have toxic effects on the microbial community and important bacterial groups (e.g., nitrogen-fixing bacteria) (Zhang et al., 2017; Ilahi et al., 2021). The long-term use of inorganic fertilizers can pollute water (Khan et al., 2018). Fertilizer leaching results in the eutrophication of aquatic and freshwater bodies (Zhang et al., 2017; Khan et al., 2018).

Plant growth-promoting rhizobacteria (PGPR) are soil bacteria that colonize plant rhizospheres and increase their growth, thus constituting a powerful tool in agriculture (De-Bashan et al., 2012; Gouda et al., 2018; Santoyo et al., 2021). They stimulate plant growth by fixing atmospheric nitrogen (N_2_) and solubilizing potassium (K) and phosphate (P) through organic acid and enzyme secretion. Microorganisms enhance the accessibility of various forms of recalcitrant P in soils (Liang et al., 2020) through the action of acid phosphatase (encoded by olpA), alkaline phosphatase (phoD, phoA), phytase (appA), phosphonatase (phnX) and CP lyase (phnJ) that can release orthophosphate to make them available to plants (Liang et al., 2020). Additionally, they secrete a variety of organic acids (tricarboxylic cycle acid, glycolic acid, malonic acid, formic acid, propionic acid, oxal, lactic acid, acetic acid, tartaric acid) that generate rhizosphere acidification and enhance phosphorus and potassium solubilization of minerals (Pan and Cai, 2023).

Additionally, PGPR release phytohormones that act as plant growth regulators, including indole acetic acid (IAA) and cytokinins (De-Bashan et al., 2012; Gouda et al., 2018; Panchami et al., 2020). These hormones increase root biomass allowing a greater uptake of nutrients from the soil. Furthermore, PGPR secrete enzymes that degrade ethylene or γ-aminobutyric acid (GABA), allowing the plant to have greater tolerance for biotic and abiotic stress (De-Bashan et al., 2012; Gouda et al., 2018; Panchami et al., 2020). The rational use of fertilization, together with PGPR inoculation, could contribute to the optimization of inorganic fertilizer use.

Among the PGPR, there is great scientific and economic interest in the *Bacillus* genus because it is genetically diverse and widely distributed across various ecological niches (Altimira et al., 2022). It can form spores, which makes it a stable bioinoculant in the soil (Cho and Chung, 2020). Crops treated with *Bacillus* spp. exhibit plant growth improvements associated with changes in community structure. Quin et al. (Qin et al., 2017) demonstrated that treatment with the strain *Bacillus* sp. L-S60 significantly improves cucumber seedling growth and the bioavailability of macronutrients (K and P). This change is associated with an increase in the abundance of PGPR genera in the rhizosphere. In contrast, the addition of *Proteus vulgaris* strain JBLS202 promotes the growth of kimchi cabbage and the upregulation of genes involved in nitrogen cycling, indicating changes in the ecological function of the rhizosphere soil (Bhattacharyya et al., 2018). These changes are associated with an increase in Proteobacteria, Acidobacteria, and Actinobacteria in the treated rhizosphere compared to the control.

The currently commercialized plant biostimulant products based on *Bacillus* are RhizoVital (*Bacillus velezensis* FZB42; ABiTEP, GmbH, Berlin, Germany), Serenade (*B. velezensis* QST713, Bayer, Leverkusen, Germany), Amylo-X WG (*Bacillus amyloliquefaciens* subsp. *plantarum* D747; Certis Europe BV, Utrecht, The Netherlands), RhizoPlus (*Bacillus subtilis* FZB24; ABiTEP), Sonata (*Bacillus pumilus* QST2808; AgraQuest, Davis, CA, USA), and Taegro (*B. subtilis* var. *amyloliquefaciens* FZB24; Novozymes Biologicals, Salem, VA, USA) (Altimira et al., 2022). In addition to the strains described, we characterized the *Bacillus safensis* RGM 2450 and *Bacillus siamensis* RGM 2529 strains isolated from the rhizospheres of cardamom crops and Guatemala native forest, respectively. Tomato seeds inoculated with a combination of both strains showed synergistic activity in stimulating seedling growth and hydration (Altimira et al., 2022).

Considering the necessity for farmers to optimize fertilizer use due to a 55.61% increase in fertilizer prices over the past 5 years (yCharts, 2024) and the growing concern of the community regarding food safety and environmental pollution, the objectives of this study were to evaluate the effects of inoculating formulations of *B. safensis* RGM 2450 and *B. siamensis* RGM 2529 on the productivity and rhizosphere bacterial community of tomato plants under a reduced fertilization program in a greenhouse. This is the first report demonstrating that the application of a formulation based on a mixture of *B. siamensis* and *B. safensis* in a fertilization program reduced by 33% is equivalent in productivity to a conventional fertilization program for tomato cultivation, achieving an increase in potential plant growth-promoting rizobacteria of the genus *Flavobacterium*.

## 2 Materials and methods

### 2.1 Strain and medium

The strain *B. safensis* RGM 2450 was isolated from a cardamom cultivation rhizosphere in Cubilhuitz (15°45′59′′, 90°30′04′′), Alta Verapaz, Guatemala. The strain *B. siamensis* RGM 2529 was isolated from a native forest rhizosphere in San Vicente Pacaya (14°24′08′′, 90°36′42′′), Escuintla, Guatemala (Altimira et al., 2022). Both strains were maintained at the Bank of the Chilean Collection of Microbial Genetic Resources at the Agricultural Research Institute (INIA, acronym in Spanish) (Altimira et al., 2022). These strains were routinely cultured in Luria-Bertani (LB) medium at 30°C.

### 2.2 Wettable powder formulations of a *Bacillus* mixture based on *Bacillus safensis* RGM 2450 and *Bacillus siamensis* RGM 2529

Suspensions of *B. safensis* RGM 2450 and *B. siamensis* RGM 2529 were grown in LB medium at 30°C for 18 h and were used to inoculate 7 L stirred tank reactors (ez-Control, Applikon Biotechnology, Delft, Netherlands) containing 5 L of LB medium. The aerated cultures were stirred (200 to 800 rpm) and supplied with 1 vvm of air for 24 h at 28°C. Subsequently, the cultures were centrifuged at 5000 × g for 5 min, washed three times, and resuspended in 0.9% NaCl. The RGM 2450 and RGM 2529 strains were blended in a 1:1 ratio and formulated as a WP as described by Altimira et al. (2021). The number of viable cells in this formulation was 2 × 10^8^ CFU/g.

### 2.3 Tomato seed inoculation

For the inoculation, 100 seeds of tomato variety Roma VF were disinfected with 2% sodium hypochlorite for 3 min and then washed five times with sterile distilled water to remove the disinfectant. Subsequently, the seeds were incubated under agitation for 40 min with the following treatments: (1) 50 seeds were embedded in a bacterial suspension (10^9^ CFU/mL) of strains *B. safensis* RGM 2450 and *B. siamensis* RGM 2529 in a 1:1 ratio; (2) 50 seeds were embedded in a *Bacillus velezensis* strain QST713 resuspension (10^9^ CFU/mL) from a commercial product; and (3 and 4) 50 seeds were embedded in 0.9% NaCl and separated into two groups of 25 seeds for subsequent treatments. Seeds were then sown superficially in a peat/perlite/compost substrate (composition 2:1:1) in seedling trays 110 mm in depth and 5 × 5 cm in surface area and placed in a greenhouse. The nutritional characteristics of the substrate were: organic matter content, 42.8%; total N, 1.38%; total P, 0.28%; total K, 0.86%, pH 7.2.

### 2.4 Reinoculation of plant treatments in greenhouse conditions

The previously inoculated or uninoculated tomato seedlings were transplanted into 5 L pots with the substrate (Section 2.3). The treatments (25 plants each) were the application of a 1:1 WP formulation of RGM 2450 plus RGM 2529 treated with 66% fertilization (T1, 66% fertilization + BM), the application of a commercial biostimulant based on *Bacillus velezensis* strain QST713 treated with 66% fertilization (T2, 66% fertilization + commercial biostimulant), the application of 66% fertilization without bacterial biostimulant (T3, 66% fertilization), the application of 100% fertilization with BM (T4, 100% fertilization + BM), the application of 100% fertilization with commercial biostimulant (T5, 100% fertilization + commercial biostimulant), and the application of 100% fertilization without bacterial biostimulant (T6, 100% fertilization). The inorganic fertilizer was applied according to the manufacturer’s instructions (Multicote 8, ANASAC, Chile) and the fertilization percentage was selected for each treatment. Multicote 8 is a polymer that allows controlled release of NPK. It is composed of 17% N (4,5% NH_4_, 4,5% NO_3_, and 8% NH_2_), 17% K (K_2_O), and 17% P (P_2_O_5_). In addition, 60 days after transplanting, the plants in treatments T1, T2, T4, and T5 were re-inoculated with 1 g of bacterial biostimulant, equivalent to 10^8^ CFU/plant, every 15 days. The biostimulant was resuspended in water and directly added to the rhizosphere. All the treatments received four applications of 0.8 g per plant of Ultrasol Calcium (SQMC, Chile) every 15 days. The experiment consisted of six treatments, each with 25 repetitions (25 pots). One experimental unit corresponded to a plant grown in a pot. The assay was carried out in a biosafety greenhouse located in experimental station of the agricultural research institute, Santiago, Chile. Plants were subjected to automatic irrigation at a controlled temperature of 25°C, photoperiod of 18:6 h, and 40% RH.

### 2.5 Macronutrient measurements in vegetable tissue

Plants were harvested 27 weeks after transplanted. Samples of leaves, stems, and fruits from each treatment were washed with water and dried at 70°C to constant weight. Afterward, they were ground and sieved through a 1 mm pore size sieve. The sieved samples were then digested with sulfuric and salicylic acids (to avoid loss of nitrate) at 100°C for 2h and selenium (as a catalyst for the reaction). Subsequently, it was digested with hydrogen peroxide at 280°C for at least 1h which oxidized most of the organic matter. Excess hydrogen peroxide was removed, and the samples were filtered to determine the NPK concentration. The total N content of the samples was determined according to the Kjeldahl method (Kalra, 1998; ISO 5725, 2019). The sample digestion converts nitrogen in the sample to ammonium sulfate. The sampled digested was neutralized by the addition of NaOH, which converted the ammonium sulfate to ammonia. Then it was distilled off and titrated with HCl. The P concentration was determined using the vanado-molybdo phosphoric acid yellow method (Kalra, 1998; ISO 5725, 2019). Each digested sample was mixed with nitro-vanadate-molybdate solution. The absorbance of the colored P-vanadiomolidate complex formed was measured at 466 nm. The K concentration was determined using atomic emission spectrophotometry with air-acetylene flame by direct aspiration. The emission of the samples was measured at 766 nm. Ionization interferences of potassium are minimized by adding lanthanum (Kalra, 1998; Temminghoff and Houba, 2004; ISO 5725, 2019). Finally, the values resulting from each determination were normalized as a percentage of the value obtained from the tissue by summing all values (leaves, stems, and fruits) in each treatment to determine the distribution of macronutrients in every section analyzed.

### 2.6 Evaluation of tomato parameters

The following parameters were measured for tomatoes harvested in each treatment: (1) average wet weight, (2) average caliber (equatorial diameter), and (3) percentages of macronutrients normalized to the average dry weight of harvested tomatoes. The tomato wet weight and caliber from each treatment was recorded using a analytical balance and meter ruler. The percentage of macronutrients normalized to the average dry weight of harvested tomatoes was obtained with methodology described in section 2.5.

### 2.7 Microbiome analysis

The diversity and structure of the rhizosphere were compared among treatments. The three replicates of these treatments were mixed, yielding two composite samples per treatment. DNA was extracted from the samples using the DNeasy Power Soil Kit (QIAGEN, Germany). The concentration of genomic DNA was quantified using a fluorometer following the manufacturer’s instructions (Qubit, Invitrogen, CA, USA). Subsequently, the DNA was sent to MACROGEN for V3-V4 hypervariable region amplification of the 16S rRNA genes, library generation, and sequencing (2 × 250 base pairs, PE250) using the HiSeq2500 platform (Illumina, CA, USA).

The quality of the raw 16S reads was evaluated using FASTQC v.0.11.9. After quality control, microbial amplicon sequence variants (ASVs) were identified for each sample using DADA2 v1.8 package in R (Callahan et al., 2016), which effectively performs quality filtering, error calculation, and chimeric sequence removal before ASV identification. The ASVs were then taxonomically classified using the RDP Classifier against the Silva rRNA gene database v128 with a confidence threshold of 80%.

Alpha diversity indices (Shannon and Simpson) were calculated for all libraries using the Microbiome v1.19.1 package in R (Lahti and Sudarshan, 2017) and statistical differences were determined by a multiple t-test. To construct the microbial composition graphs, phyloSeq object abundances were transformed into relative abundances. The 21 most abundant genera were selected, including those not assigned taxa (“unknown”), and reordered according to their phylum to enhance the clarity of the graphs. These plots were created using the Microbiome R software package. The samples were categorized based on the biostimulant applied.

### 2.8 Colonization of microorganisms in tomato roots

Treatments T1 and T2 were selected to evaluate bacterial colonization on the surface of tomato roots in comparison with the T3 treatment. The roots from these treatments were extracted and washed manually with abundant water to avoid damaging the root structure. Scanning electron microscopy (SEM) analysis of the root samples was performed by fixation of the samples with 3% glutaraldehyde in 0.268 M sodium cacodylate buffer (pH 7.0), followed by dehydration and critical point drying, and finally by gold-coating on a 0.22 mm poly-carbonate membrane. The samples were visualized using TM 3000 SEM (Hitachi, Tokyo, Japan).

### 2.9 Statistical analysis

Based on a random distribution of the treatments in the greenhouse (section 2.4), we carried out the ANOVA LSD Fisher test (α = 0.05) to compare the values of tomato average wet weight, the average weight per harvest, average caliber, and percentages of macronutrients normalized to the average dry weight of harvested tomatoes. The results were analyzed and graphed using Statgraphics Centurion XVI (Statgraphics Technologies, USA) and GraphPad Prism 9 (2020 version). To determine significant differences between the alpha diversities of the treatments, the Wilcoxon test was performed using R version 4.2.1. Statistical significance was set at P < 0.05. significant. Finally, the samples were categorized based on the applied biostimulant, and a t-test was performed to identify statistically significant differences in the relative abundance of taxa at the genus level. All statistical analyses were performed using the GraphPad Prism software v8.0.1 (GraphPad Software LLC, San Diego, CA, USA; https://www.graphpad.com/).

## 3 Results

### 3.1 Macronutrient contents in the leaf, stem, and tomato

**Figure 1:**
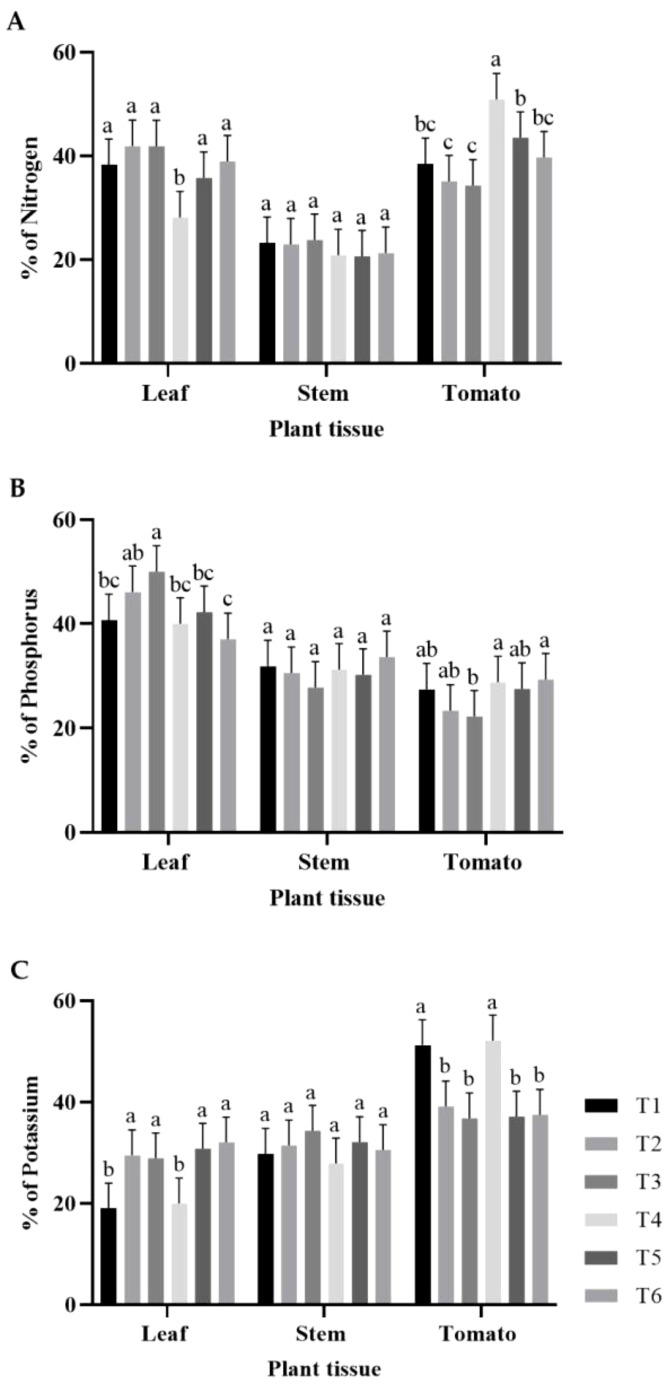
Percentages of macronutrients in leaves, stems, and tomatoes: **(A)** percentage of N; **(B)** percentage of P; **(C)** percentage of K. The bars represent the average percentage for each treatment. The whiskers represent the standard deviation, and the letters represent the results of the LSD test (∝=0.05). T1, 66% fertilization + BM; T2, 66% fertilization + commercial biostimulant; T3, 66% fertilization; T4, 100% fertilization + BM; T5, 100% fertilization + commercial biostimulant; T6, 100% fertilization.

The N, P, and K concentrations were determined in plant tissues from each treatment (**Figure 1**). Leaf N concentrations in the treatments were similar, with no statistically significant differences, except for in T4. (**Figure 1A**). This treatment resulted in the lowest leaf N concentration. For stem N concentration, all treatments showed similar percentages without statistically significant differences (**Figure 1A**). Additionally, regarding tomato N concentration, T4 showed the highest N content, followed by groups T5, T6, and T1, and finally by groups T2 and T3 (**Figure 1A**). Regarding the P concentration in the leaves, T3 had the highest percentage, followed by T2, T1, T4, T5, and T6. The percentage of P in the stems was similar in all treatments, but the differences were not statistically significant. In contrast, the P concentration in tomato was the highest in T4 and T6, followed by groups T1, T2, T5, and finally T3 (**Figure 1B**). Regarding K concentration in the leaves, the groups composed of T2, T3, T5, and T6 were significantly higher than the groups composed of T1 and T4. In the stems, no statistical differences were observed between treatments. For tomatoes, groups T1 and T4 had the highest percentages. The other treatments did not show statistically significant differences, with the lowest percentages (**Figure 1C**).

### 3.2 Weight, caliber, and percentage of nutrients in tomato

**Figure 2:**
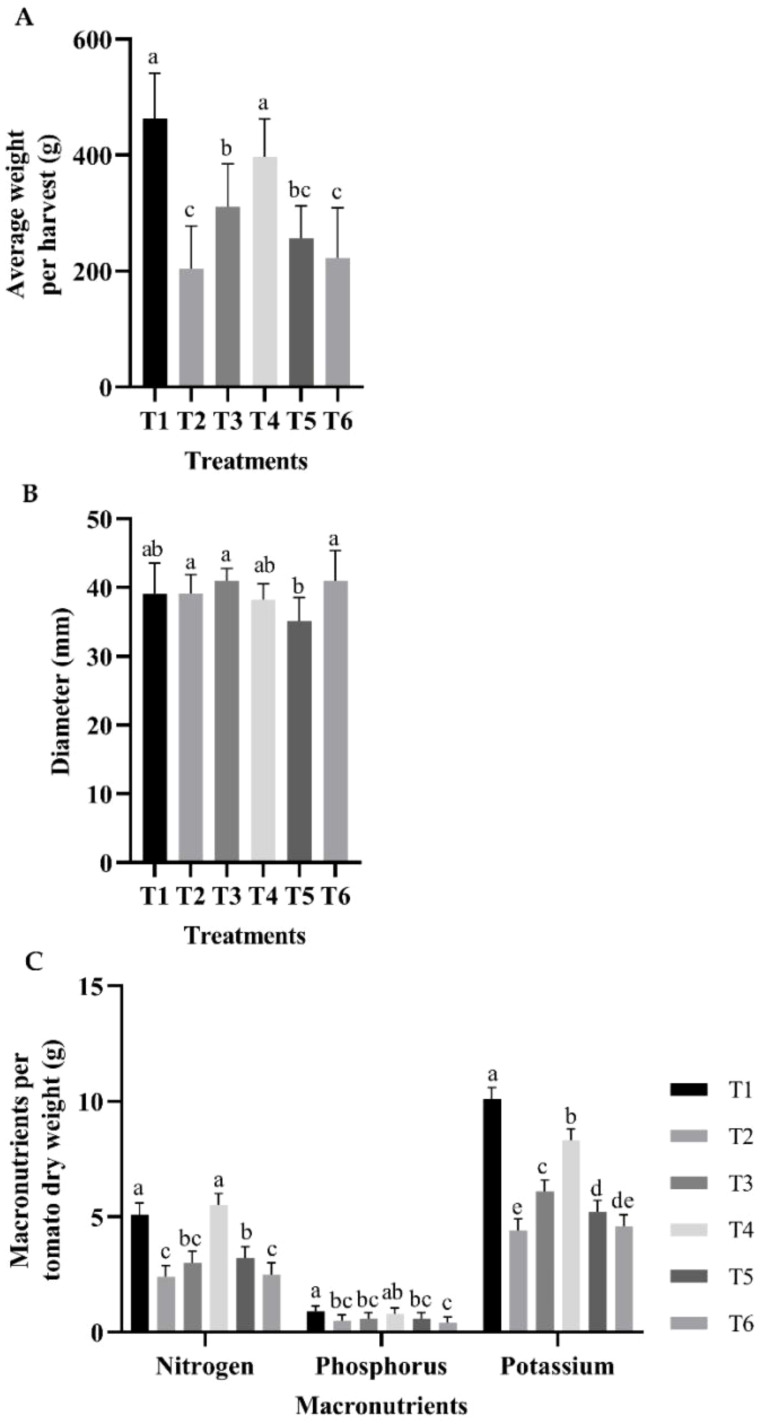
Evaluation of fruit: **(A)** tomato average weight per harvest; **(B)** average diameter; **(C)** percentages of macronutrients normalized to the average dry weight of tomatoes harvested from each treatment. The bars represent the average of each treatment. The whiskers represent the standard error, and the letters represent the results of the LSD test (∝=0.05). T1, 66% fertilization + BM; T2, 66% fertilization + commercial biostimulant; T3, 66% fertilization; T4, 100% fertilization + BM; T5, 100% fertilization + commercial biostimulant; T6, 100% fertilization.

The T1 and T2 treatments produced the highest average tomato weight per harvest (**Figure 2A**). Regarding caliber, treatments T2, T3, and T6 showed no statistical difference, followed by the group composed of T1 and T4, and finally T5 (**Figure 2B**). However, the percentages of macronutrients normalized to the average dry weight of tomatoes harvested from each treatment showed in the case of N, treatments T4 and T1 had the highest values with a significant difference in comparison to T3, followed by T5, and finally by T2 and T6. In the case of P, the highest value was obtained at T1, followed by T4, and the group was composed of T2, T3, T5, and T6. Finally, in the case of K, the highest value was obtained for T1, followed by T4, T5, and T3, with statistically significant differences between them, followed by T2 and T6 (**Figure 2C**).

### 3.3 Microbiome analysis

**Figure 3:**
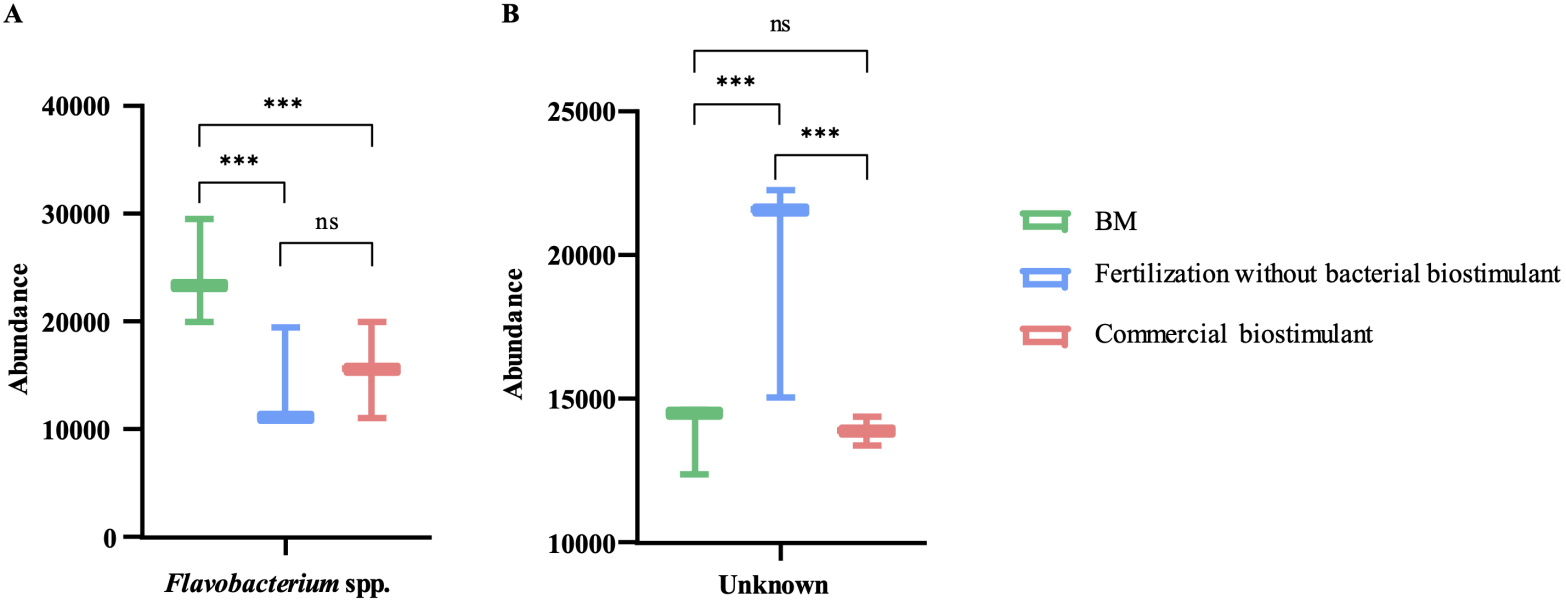
Genus level comparison among treatments. Comparison of relative abundance of Flavobacterium spp. and unknown genus in treatment supplemented with BM, only fertilization, or commercial biostimulant. Statistical analysis was performed using the Kruskal-Wallis test. ns, not significant; ***, P<0.001.

**Figure 4:**
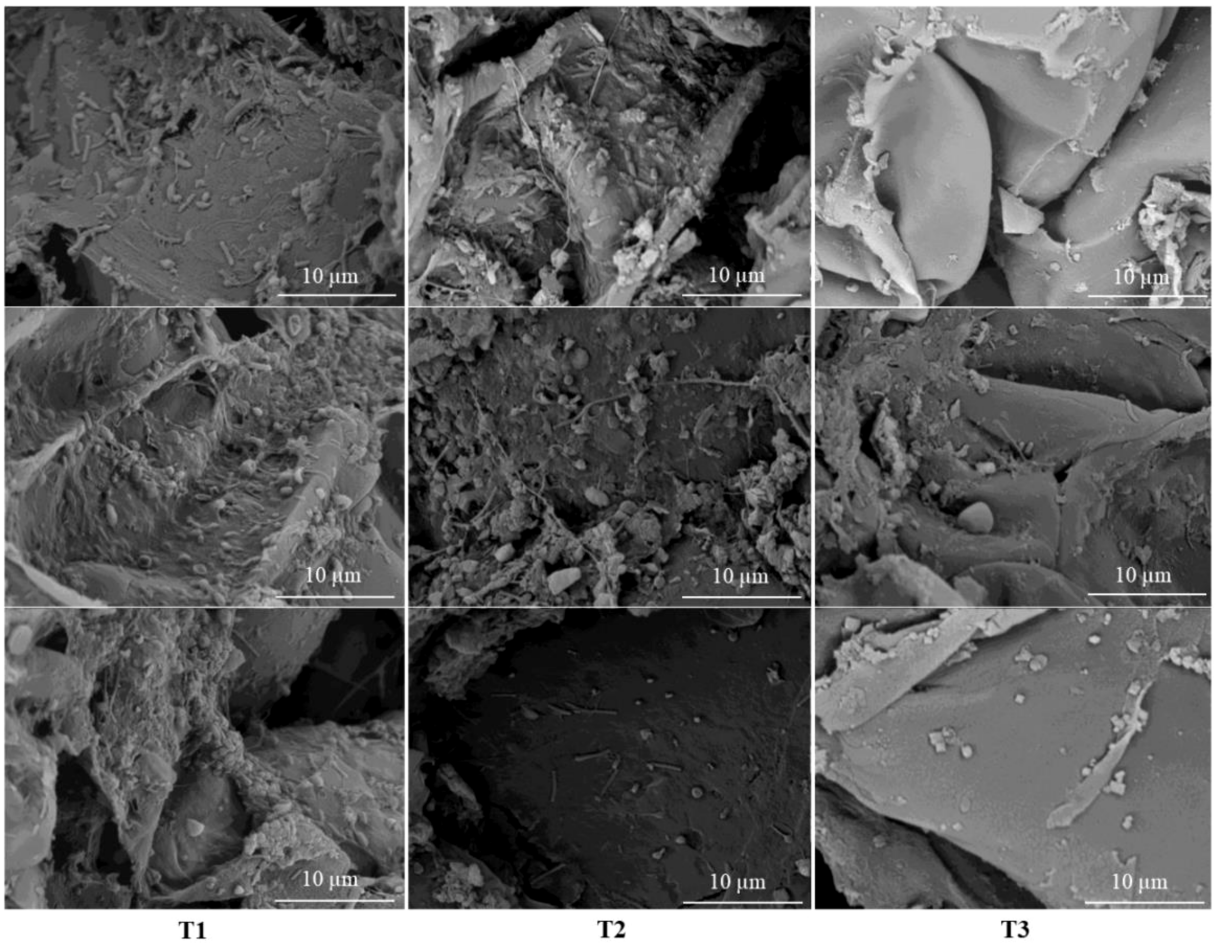
Scanning electron microscopy (SEM) images of the tomato root surface from treatments with 66% fertilization with and without biostimulant inoculation: T1, 66% fertilization + BM; T2, 66% fertilization + commercial biostimulant; T3, 66% fertilization.

The treatments showed no significant differences in Simpson’s or Shannon’s diversity indices using 16S rRNA V3-V4 hypervariable region analysis (**Table 1**). This result suggests that the incorporation of bacterial biostimulant at 10^8^ CFU/plant and/or increasing fertilizer concentration from 6 to 100% in the substrate did not significantly alter rhizosphere diversity. Additionally, rhizosphere analysis indicated a significant increase in the abundance of microorganisms belonging to the genus *Flavobacterium* in the BM-supplemented treatment with the fertilization regimen, with or without commercial biostimulants (**Figures 3**, **4**). In contrast, a significant reduction of unknown genera abundance was observed with the bacterial biostimulant treatment.

**Table 1:** Rhizosphere alpha diversity indices of the *Bacillus* bacteria and control treatments.

Treatment	Alpha diversity	
	Simpson	Shannon
T1, Fertilization 66% + BM	0,934 ± 0,003a	4,678 ± 0,138a
T2, Fertilization 66% + Commercial biostimulant	0,926 ± 0,002a	4,526 ± 0,09a
T3, Fertilization 66%	0,934 ± 0,003a	4,678 ± 0,138a
T4, Fertilization 100% + BM	0,910 ± 0,012a	4,409 ± 0,101a
T5, Fertilization 100% + Commercial biostimulant	0,977 ± 0,010a	5,248 ± 0,15a
T6, Fertilization 100%	0,940 ± 0,025a	4,755 ± 0,015a

Different letters in each treatment represent a statistically significant difference.

### 3.4 Tomato root bacterial colonization

The T1 and T2 treatments were selected to evaluate bacterial colonization on the surface of tomato roots in comparison to the T3 treatment using SEM (**Figure 4**). The treatments with 66% fertilization and the addition of bacterial biostimulants (T1 and T2) showed a markedly higher bacterial cell count and the presence of bacterial biofilms on root surfaces as compared to the T3 treatment with the same percentage of fertilization but without bacterial biostimulant addition.

## 4 Discussion

An increasing demand for vegetables has led farmers to grow these crops extensively (Masood et al., 2020) and has foster greenhouse cultivation adoption with the aid of expensive chemical fertilizer to ensure high year-round production. Therefore, improving nutrient use efficiency to increase crop yields is a goal that many agricultural scientists are working toward, as it would save natural resources and reduce the impact of inorganic fertilization on the environment. One strategy to achieve this is the incorporation of PGPR into agricultural soil. *B. safensis* RGM 2450 and *B. siamensis* RGM 2529 are PGPR that have previously shown synergistic activity in stimulating tomato seedling growth (Altimira et al., 2022).

In the present study, the addition of these formulated strains, RGM 2450 and RGM 2529, to tomato crops subjected to 66% (T1) and 100% (T4) fertilization programs resulted in a significantly higher percentage of NPK in the fruit (**Figures 1**, **2C**). Both treatments had a lower percentage of these elements in the stems and leaves than the other treatments (**Figures 1A, B**). These results indicate that the addition of PGPR strains RGM 2450 and RGM 2529 promoted the mobilization of these nutrients in the vegetable tissue, along with a higher tomato weight per harvest in T1 and T4 (**Figure 2A**). In particular, the highest K percentage among all treatments was found in tomatoes in the T1 and T4 treatments. This element is especially important because it is involved in tomato quality parameters, such as fruit size, soluble solids, lycopene, and vitamin C (Cruz et al., 2018). The potential mechanisms that support these results lies in the metabolic capabilities encoded in the genomes of the RGM 2450 and RGM 2529 strains. Both strains encode enzymes in their genomes that participate in the production of organic acids, including gluconic acid and 2-ketogluconic acid, which contribute to the solubilization of insoluble P and K salts (Altimira et al., 2022). Additionally, both strains possess the phoA gene, which encodes an alkaline phosphatase involved in P solubilization (Altimira et al., 2022). This enzyme splits phosphate groups into organic compounds, increasing their bioavailability. In addition, the B. siamensis RGM 2529 genome encodes a phytase (Altimira et al., 2022). The enzyme sequentially cleaves six orthophosphate groups attached to the phytate inositol molecule. The *B. siamensis* RGM 2529 genome contains a gene cluster that encodes a urease, nitrite, and nitrate reductase involved in converting urea into ammonia, nitrate into nitrite, and nitrite into ammonia, respectively (Altimira et al., 2022). The availability of both nitrate and ammonium stimulates plant growth beyond that observed with either N source alone (Britto and Kronzucker, 2002; Hachiya and Sakakibara, 2017). On the other hand, in the study of Masood et al. (2020) the inoculation of *Bacillus pumillus*, phylogenetically close to *B. safensis*, increased the rhizobacterial population, the expression of the bacterial *nifH* gene (involved in nitrogen fixation) and the soil nitrogenase activity within a fertilization program (supply 150 mg N kg-1 dry soil in the form of urea) of tomato plants leading to improve the tomato growth and N uptake, suggesting that the inoculation of *B. pumilus* improves the growth of tomato under due to an increase in N uptake by roots from *B. pumilus*-assisted fixed N in soil (Masood et al., 2020). Although such a statement or inference could not be drawn from our study, the assessment of nitrogenase activity in soils inoculated with *Bacillus safensis* and *Bacillus siamensis* should be considered in future research.

Other studies on tomatoes using phosphate-solubilizing bacteria (PSB) have shown significantly higher dry weight and total N and P in shoots than in control treatments (without PSB) (Kim et al., 1998; Walpola and Yoon, 2013). Additionally, the application of a biofertilizer containing three PGPR and mycorrhizal fungi significantly increased plant growth and uptake of N and P in maize, and also improved soil organic matter and total N in the soil (Wu et al., 2005).

In our study, T1 and T4 showed the highest average tomato weight per harvest (**Figure 2A**). These results could be explained by the fact that the *B. safensis* RGM 2450 and *B. siamensis* RGM 2529 strains possess growth-promoting compound biosynthetic pathways such as IAA, zeatin, acetoin, 2,3-butanediol, and polyamines (Altimira et al., 2022). These hormones stimulate the growth of plant organs via cell division and expansion, increasing the surface area for nutrient absorption (Arikan and Pirlak, 2016).

Consistent with these results, applications including *Pseudomonas* and *Bacillus* strains stimulate growth and increase yield in pepper and tomato (Dursun et al., 2010), spring barley (Şahin et al., 2004), apricot (Esitken et al., 2003; Altindag et al., 2006), apple (Aslantaş et al., 2007), sugar beet (Çakmakçı et al., 2001; Şahin et al., 2004), and sour cherry (Arikan and Pirlak, 2016). Additionally, Adesemoye et al. (2009) reported that a mixture of the PGPR strains *B. amyloliquefaciens* IN937 and *B. pumilus* T4 in soil at a 75% fertilizer rate produced a tomato yield similar to that at a 100% fertilizer rate. This study demonstrates the relevance of bacterial biostimulants as an agricultural tool to reduce the application of inorganic fertilizers.

**Figure 5:**
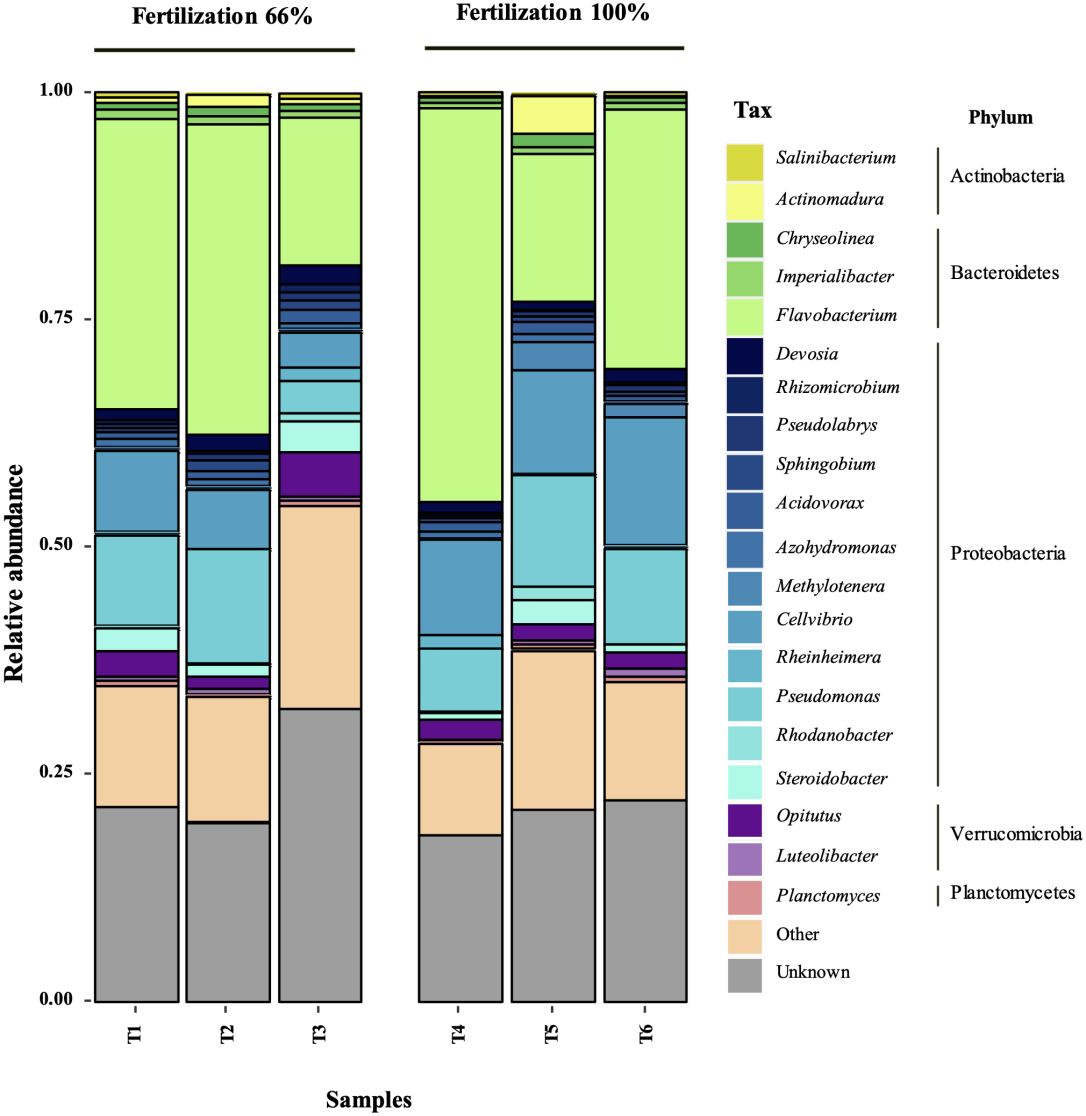
Relative abundance of the most abundant bacterial genera in the rhizosphere of the treatments. The 21 most abundant genera were selected, including those not assigned taxa (“unknown”). In each plot, the genera are grouped by phylum. T1, Fertilization 66% + BM; T2, Fertilization 66% + Commercial biostimulant; T3, Fertilization 66%; T4, Fertilization 100% + BM; T5, Fertilization 100% + Commercial biostimulant; T6, Fertilization 100%.

In addition, PGPR inoculation can enhance the NPK (nitrogen, phosphorus, potassium) content of soils by altering the soil microbial community (Masood et al., 2020; Pan and Cai, 2023). PGPR presence in the soil modifies the diversity of the microbial community and increases the activity of associated enzymes, thereby changing the NPK content of the soil and promoting crop growth and yield (Masood et al., 2020; Pan and Cai, 2023). In this study, the RGM 2450 and RGM 2529 supplementation of the substrate significantly increased the abundance of *Flavobacterium* in the rhizosphere (**Figures 3A**, **5**). Consistently, in the study by Wang et al. (2023) the inoculation of the PGPR strain, *B. velezensis* BER1 altered the tomato rhizosphere microbiome with an overrepresentation of the Flavobacterium bacteria. This genus represents an important fraction of the rhizosphere microbiome in tomatoes (Kolton et al., 2016; Seo et al., 2024), bell peppers (Graber et al., 2010), lettuce (Cardinale et al., 2015), peanuts (Haldar et al., 2011), maize (Li et al., 2014), and *Arabidopsis thaliana* plants (Bulgarelli et al., 2012; Lundberg et al., 2012; Bodenhausen et al., 2013). Their abundance in the rhizosphere is positively correlated with plant biomass (Bulgarelli et al., 2012), plant resistance to pathogens (Niroshini Gunasinghe and Karunaratne, 2009; Manter et al., 2010; Lundberg et al., 2012), phytohormone production (Kolton et al., 2016), and stimulation of fruit ripening (Sang and Kim, 2012). Additionally, *Flavobacterium* species has been involved in supply essential macro- and micronutrients to their host plants (Youseif, 2018; Zhang et al., 2021; Choi et al., 2023; Seo et al., 2024). For instance, *Flavobacterium* sp. R6S-5-6 can enhance nitrogen availability for the host plant by activating genes involved in nitrogen fixation, including the nif gene (*nifU*), fix gene (*fixF*), and the global nitrogen regulator (ntcA) (Choi et al., 2023). Additionally, phosphorus solubilization facilitated by *Flavobacterium*-specific alkaline phosphatase PhoX and PafA can improve plant tolerance to abiotic stress, promote plant growth, and assist in overcoming phosphorus deficiency (Youseif, 2018; Lidbury et al., 2021; Zhang et al., 2021; Choi et al., 2023; Seo et al., 2024). These reports suggest that the increased abundance of *Flavobacterium* spp. in the rhizosphere by the addition of strains the RGM 2450 and RGM 2529 treatments could have contributed to the significant increase in the total weight of the tomatoes harvested and increased bioavailability of N and P in these treatments. In contrast, treatments with biostimulant bacteria showed a significant reduction in unknown genera compared to treatments with fertilization alone. This result suggests that biostimulant bacteria promote the abundance of genera/species previously known for some characteristics or described roles in some ecosystems.

Additionally, the effects of the PGPR inoculum on tomato roots were evaluated using SEM. Treatments T1 and T2 showed the presence of bacterial biofilms on the root surfaces (**Figures 4A, B**), whereas a scarce presence of bacteria on the root surfaces was observed in T3 (without bacteria) (**Figure 4C**). These results suggest that inoculation with RGM 2450 and RGM 2529 changed the microbial community structure of the rhizosphere of tomato seedlings, resulting in an increased abundance of certain plant growth-promoting bacteria in the soil. Future evaluations of yield and fruit nutritional quality will contribute to confirming the effects of inoculation programs with RGM 2450 and RGM 2529 formulations under a reduced fertilization program in tomato crops under field conditions.

## 5 Conclusion

The increasing demand for vegetables has encouraged greenhouse cultivation to ensure high yields and quality of fresh produce throughout the production year. This has led to a high demand for fertilizers with their potential ecological and economic impact. To mitigate these consequences the inoculation of a biostimulant formulation consisting of a mixture of *B. safensis* RGM 2450 and *B. siamensis* RGM 2529 under a reduced inorganic fertilization program resulted in increased tomato production and enhanced rhizosphere colonization, concomitant with a higher level of PGPR genes in the rhizosphere bacterial community structure in a greenhouse trial. Therefore, the incorporation of a biostimulant in combination with 66% inorganic fertilization is a sustainable approach to obtaining good tomato yield and reducing the environmental impact of inorganic fertilization.

## Data Availability

The microorganisms Bacillus safensis RGM 2450 and Bacillus siamensis 2529 used in this study are deposited in the INIA microbial genebank (https://www.cchrgm.cl/nosotros-1/). In the supplementary information of Altimira et al., 2022 you can find the information of the genomes and metabolic pathway recontructions. Finally, the sequence data from this study have been deposited with the GenBank Data Libraries (NCBI) under BioProject PRJNA1148014.
